# Denitrification contributes to N_2_O emission in paddy soils

**DOI:** 10.3389/fmicb.2023.1218207

**Published:** 2023-06-16

**Authors:** Hua Xiang, Yiguo Hong, Jiapeng Wu, Yu Wang, Fei Ye, Jiaqi Ye, Jing Lu, Aimin Long

**Affiliations:** ^1^State Key Laboratory of Tropical Oceanography (LTO), South China Sea Institute of Oceanology, Chinese Academy of Sciences, Guangzhou, China; ^2^Key Laboratory for Water Quality and Conservation of the Pearl River Delta, Institute of Environmental Research at Greater Bay Area, Ministry of Education, Guangzhou University, Guangzhou, China; ^3^University of Chinese Academy of Sciences, Beijing, China

**Keywords:** denitrification, N**_2_**O emission, functional gene, microbial taxa, paddy soils

## Abstract

Denitrification is vital to nitrogen removal and N_2_O release in ecosystems; in this regard, paddy soils exhibit strong denitrifying ability. However, the underlying mechanism of N_2_O emission from denitrification in paddy soils is yet to be elucidated. In this study, the potential N_2_O emission rate, enzymatic activity for N_2_O production and reduction, gene abundance, and community composition during denitrification were investigated using the ^15^N isotope tracer technique combined with slurry incubation, enzymatic activity detection, quantitative polymerase chain reaction (qPCR), and metagenomic sequencing. Results of incubation experiments showed that the average potential N_2_O emission rates were 0.51 ± 0.20 μmol⋅N⋅kg^–1^⋅h^–1^, which constituted 2.16 ± 0.85% of the denitrification end-products. The enzymatic activity for N_2_O production was 2.77–8.94 times than that for N_2_O reduction, indicating an imbalance between N_2_O production and reduction. The gene abundance ratio of *nir* to *nos*Z from qPCR results further supported the imbalance. Results of metagenomic analysis showed that, although Proteobacteria was the common phylum for denitrification genes, other dominant community compositions varied for different denitrification genes. Gammaproteobacteria and other phyla containing the *nor*B gene without *nos*Z genes, including Actinobacteria, Planctomycetes, Desulfobacterota, Cyanobacteria, Acidobacteria, Bacteroidetes, and Myxococcus, may contribute to N_2_O emission from paddy soils. Our results suggest that denitrification is highly modular, with different microbial communities collaborating to complete the denitrification process, thus resulting in an emission estimation of 13.67 ± 5.44 g N_2_O⋅m^–2^⋅yr^–1^ in surface paddy soils.

## 1. Introduction

Nitrous oxide (N_2_O) is not only a significant ozone-depleting substance (Ravishankara et al., 2009), but is also a well-known greenhouse gas with strong radiative forcing effects (Zumft and Kroneck, 2007). Since the industrial revolution, the concentration of N_2_O has increased at an annual rate of 0.25%, and the atmospheric N_2_O concentration has reached 331 ppb (Solomon et al., 2007). Moreover, the greenhouse effect of N_2_O is 310 times higher than that of the equivalent carbon dioxide (Lashof and Ahuja, 1990). Thus, N_2_O has received increasing attention owing to the environmental problems that it may cause.

Agricultural fields that receive substantial amounts of nitrogen fertilizers are typically known as N_2_O emission hotspots (Syakila and Kroeze, 2011). According to predictions, by 2030, agricultural soils will become the primary source of N_2_O emission, contributing 59% of the total N_2_O emission released into the atmosphere (Hu et al., 2015). Although nitrification, denitrification, and nitrate dissimilation to ammonium can all generate N_2_O, denitrification is the primary pathway for N_2_O emission in terrestrial ecosystems (Sanford et al., 2012; Butterbach-Bahl et al., 2013; Harris et al., 2021). Furthermore, in the denitrification process, N_2_O exists as an intermediate product and can be both generated and consumed, leading to the emission of N_2_O being regulated by multiple functional genes (Zumft, 1997) During denitrification, NO_3_^–^ is successively reduced to NO_2_^–^, NO, N_2_O, and finally to N_2_ (Zumft, 1997). Diverse phylogenetic denitrifying bacteria contain different functional genes, including *nap*A, *nir*S, *nir*K, *nor*B, *nos*Z I, and *nos*Z II genes, which encode enzymes that complete the denitrification process (Zumft, 1997). The *nap*A gene encodes NO_3_^–^ reductase, which catalyzes the reduction of NO_3_^–^ to NO_2_^–^ (Arnoux et al., 2003). NO_2_^–^ and N_2_O reductions are considered the rate-limiting steps in denitrification (Zumft, 1997). NO_2_^–^ reduction is catalyzed by NO_2_^–^ reductases, including the *nir*S gene-encoded copper-containing NO_2_^–^ reductase and the *nir*K gene-encoded cytochrome cd1-containing NO_2_^–^ reductase (Zumft, 1997). Copper-containing or cytochrome cd1-containing NO_2_^–^ reductases are functionally identical but have different structures and catalytic sites, and generally do not coexist in one bacterial species (Coyne et al., 1989). The NO reductase (NOR), encoded by the *nor*B gene, is responsible for the reduction of NO to N_2_O (Braker and Tiedje, 2003). The N_2_O reductase (NOS) catalyzes the reduction of N_2_O, converting the greenhouse gas N_2_O into relatively harmless N_2_, thereby reducing its contribution to the greenhouse effect. NOS, encoded by either *nos*Z I or *nos*Z II genes, can complete denitrification or N_2_O reduction only (Zumft and Kroneck, 2007). The *nos*Z I and *nos*Z II genes generally do not coexist in the same bacteria, except in Thauera linaloolentis 47Lol*^T^* (Semedo et al., 2020).

Nitrous oxide is an intermediate product of the denitrification process, and N_2_O emission is typically associated with enzymes encoded by functional genes and the bacteria that harbor these genes during denitrification (Black et al., 2019). Denitrification is a complex process affected by multiple factors, including environmental parameters, the microbial composition of functional genes, and key enzymatic activities (Groffman, 2012). Rich et al. (2003) showed the community composition as well as environmental factors lead to the variation in denitrification rates in meadow and forest soils. The analysis of denitrification genes abundance can establish a stronger correlation with potential N_2_O emissions (Morales et al., 2010). For instance, the ratio of *nir* to *nos*Z can partly determine the extent of N_2_O emission in soils (Domeignoz-Horta et al., 2015) and lakes (Saarenheimo et al., 2015). However, the comprehensive mechanisms of denitrification and N_2_O emissions are yet to be elucidated.

In this study, we first investigated the dissolved N_2_O concentration in paddy water and calculated the N_2_O flux at the water–air exchange to provide background information regarding N_2_O emissions in paddy fields. Focusing on paddy soils, we performed ^15^N isotope tracer experiments to determine the potential N_2_O emission rate and calculate the end-product ratio, i.e., N_2_O/(N_2_+N_2_O). The enzymatic activities of the NOR and NOS were measured using ELISA assay kits. Subsequently, qPCR and metagenomic analysis were performed to determine the abundance and community composition of key functional genes, respectively. The aim of this study is to demonstrate the contribution and underlying mechanism of denitrification to N_2_O emission in paddy soils and provide a more comprehensive understanding of the N_2_O emission process in paddy soils.

## 2. Materials and methods

### 2.1. Sample acquisition and physiochemical property determination

**FIGURE 1:**
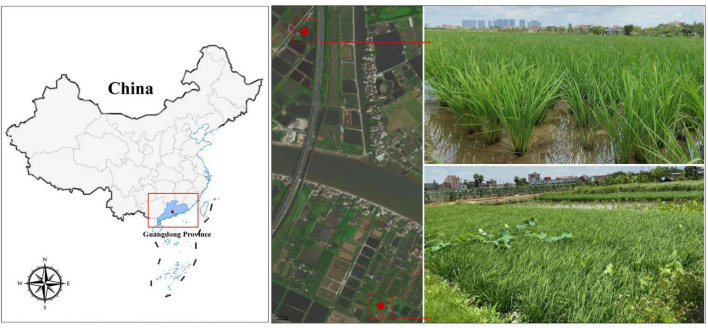
Location of Guangdong Province and sampling site of paddy fields.

The soil to be tested was obtained from two paddy fields (22°55′16″N, 113°29′24″E; 22°54′24″N, 113°29′36″E) in May 2021 from Guangdong Province, China (Figure 1). Both paddy fields had been planted with rice for many years and the paddy fields were under water-logged conditions at the time of sampling. The dissolved oxygen (DO) in the paddy water was first determined using a portable multifunctional parameter meter equipped with a DO probe (HQ40D, HACH, Loveland, CO, USA). Quickly insert a 12.5 mL vial (Exetainer, Labco Limited, Lampeter, UK) below the surface of the paddy water until the vial was overflowing and there were no air bubbles inside. The cap was immediately sealed and 200 μL of saturated ZnCl_2_ solution was added to stop microbial activities. The sample was in triplicate and stored at −4°C. The dissolved N_2_O concentration of *in situ* water was determined within 24 h. The pH level was determined using a pH meter (Mettler Toledo S220, Greifensee, Switzerland). Salinity and temperature were measured using salinometers (ATAGO, Tokyo, Japan) and geothermometers (HG04-SYQX-2, Beijing, China), respectively.

Surface soil (0–10 cm) was sampled using a sterilized shovel, and each soil sample was mixed in triplicate. Paddy soil was divided into three parts: one for measuring potential rates of N_2_ and N_2_O emissions, which was stored at 4°C; another for DNA extraction and enzymatic activity measurement, which was kept at −80°C; and the last part was stored at 4°C for pH determination and analysis of dissolved inorganic nitrogen (DIN), including NO_3_^–^, NO_2_^–^ and NH_4_^+^. DIN in the paddy soil was extracted using 2 M KCl with a soil extraction ratio of 1:5 (wt./vol.) before measurement (Bao, 2000). The DIN content was determined using the spectrophotometric method described by Wu et al. (2016) and Guan et al. (2017).

### 2.2. Dissolved N_2_O analysis

The concentration of N_2_O was measured using static headspace gas chromatography (Xu et al., 2005). The water in the vial was replaced with 5 mL of He to achieve headspace, agitated vigorously for 15 min, and stored in the dark to attain gas–liquid equilibrium. A gas chromatograph equipped with an electron capture detector (GC-2014C, Shimadzu, Tokyo, Japan) was used to measure the concentration of N_2_O (C_G_) in the headspace. The dissolved N_2_O concentration (C_L_) before gas replacement and the △N_2_O (i.e., the net increase in N_2_O) were calculated by the following equations (Lin et al., 2016):


(1) $${\text{C}}_{\text{L}}={\text{C}}_{\text{G}}\left({\text{K}}_{0}\mathrm{RT}+{\text{V}}_{\text{G}}/{\text{V}}_{\text{L}}\right)$$

(2) $$△{\text{N}}_{2}\text{O}={\text{C}}_{\text{L}}-{\text{C}}_{\mathrm{N2Oeq}}$$

where K_0_ is the equilibrium constant (Weiss and Price, 1980); R is the ideal gas constant; T is the temperature at equilibrium; V_G_ and V_L_ are the gas and liquid volumes after He replacement, respectively; and C_N2Oeq_ is the N_2_O concentration in equilibrium with the atmospheric concentration calculated based on Weiss and Price (1980).

N_2_O saturation was calculated as follows:


(3) $${\text{C}}_{\mathrm{N2Osa}}=100{\text{⋅C}}_{\text{L}}/{\text{C}}_{\mathrm{N2Oeq}}$$

The estimated N_2_O flux through the water–air interface was calculated as follows:


(4) $$\text{F}=\text{K}\cdot △{\text{N}}_{2}\text{O}$$

where K denotes the gas change rate calculated based on the method of Borges (2004).

### 2.3. Determination of potential N_2_ production rate and N_2_O emission rate

The procedures for slurry incubation were modified from Thamdrup and Dalsgaard (2002) and described in detail by Xiang et al. (2023). A mixture of fresh soil and water was pre-incubated in the dark for 72 h at a weight-to-volume ratio of 1:7. The soil slurry was then flushed with He to eliminate N_2_O and create an anaerobic environment before being transferred to vials using a syringe. The vials were separated into two groups, one group (three vials) was for measuring the concentration of remaining ^14^NO_3_^–^ such that F_n_ (the proportion of ^14^NO_3_^–^ in the total NO_3_^–^ pool after adding ^15^NO_3_^–^) can be calculated, and the other group (12 vials) was injected with ^15^NO_3_^–^ (^15^N 99.6%) to the final concentration of 100 μmol⋅L^–1^. The vials containing ^15^NO_3_^–^ were incubated for T_0_ (T_0_ = 0 h) and T_2_ (T_2_ = 2 h) at *in situ* temperature in darkness, respectively, and 200 μL of saturated ZnCl_2_ solution was injected immediately.

The ^29^N_2_ amounts (D_29_) and ^30^N_2_ amounts (P_30_) in the vials above were measured via membrane inlet mass spectrometry (HPR40, Hiden, Warrington, UK), and the potential N_2_ production rate (R_*N2*_) was calculated as follows (Xiao et al., 2018; Wu et al., 2021):


(5) $${\text{D}}_{29}={\text{P}}_{30}\text{⋅}2\text{⋅}\left(1-{\text{F}}_{\text{n}}\right){\text{⋅F}}_{\text{n}}^{-1}$$

(6) $${\text{R}}_{\mathrm{N2}}={\text{D}}_{29}+2{\text{⋅P}}_{30}$$

The N_2_O concentration in each vial was determined via a procedure similar to that used for determining the N_2_O concentration in the water sample (Xu et al., 2005). The potential N_2_O emission rates (R_*N*2*O*_) was calculated based on the dissolved N_2_O concentrations in the vials at T_0_ and T_2_ (i.e., C_*L*0_ and C_*L*2_, respectively) (Xiang et al., 2023).


(7) $${\text{R}}_{\mathrm{N2O}}=\left({\text{C}}_{\mathrm{L2}}-{\text{C}}_{\mathrm{L0}}\right)/\left({\text{T}}_{2}-{\text{T}}_{0}\right)$$

The ratio of denitrification end-products was calculated as follows:


(8) $$\text{Ratio}=100{\text{⋅R}}_{\mathrm{N2O}}/\left({\text{R}}_{\mathrm{N2}}+{\text{R}}_{\mathrm{N2O}}\right)$$

### 2.4. Measurement of enzymatic activity of NOR and NOS

The enzymatic activities of NOR (NO→N_2_O) and NOS (N_2_O→N_2_) in the soils were determined using microbial NOR and NOS ELISA kits (Yilaisa Biotechnology Co., Ltd., Jiangsu, China). Briefly, 1 g of fresh soil, standards, and HRP-labeled detection antibodies were added based on the manufacturer’s protocol. A microplate reader (BioTek Elx800, Winooski, VT, USA) was used to measure the absorbance at 450 nm. Enzymatic activity was calculated from the absorbance based on the standard curve.

### 2.5. DNA extraction and qPCR

Soil DNA was extracted using a Fast DNA Spin Kit for Soil (MP Biomedical, Irvine, CA, USA) based on the manufacturer’s protocol. The concentration and purity of the extracted DNA were verified using a NanoDrop spectrophotometer (Thermo Scientific, Wilmington, DE, USA) and agarose gel electrophoresis, respectively. Primer sets 515F/806R (Caporaso et al., 2011), cd3af/R3cd (Throback et al., 2004), F1aCu/R3Cu (Hallin and Lindgern, 1999), *nos*Z 2F/*nos*Z 2R (Henry et al., 2006), *nos*Z IIF/*nos*Z IIR (Jones et al., 2013) were used to quantify the abundance of bacterial 16S, *nir*S, *nir*K, *nos*Z I, and *nos*Z II genes, respectively. qPCR was performed using the iQ5 Real-Time PCR system (Bio-Rad, Hercules, CA, USA). The amplification procedures are present in Supplementary Table 1 and have been described previously (Xiang et al., 2022). Plasmids, samples, and negative controls were prepared in triplicate and quantified simultaneously. The specificity of the amplified products was verified via melting curve analysis and agarose gel electrophoresis. Results of the amplification efficiency (90–110%) and correlation coefficient (R^2^ > 97%) are shown.

### 2.6. Metagenomic sequencing and gene annotation

The extracted DNA was sent to Genewiz (Suzhou, China) for library construction and shotgun sequencing. The raw sequencing reads of each sample exceeded 10 Gb and were trimmed using Trimmomatic (v 0.38) (Bolger et al., 2014) to obtain high-quality clean reads and ensure the accuracy of subsequent analysis. The clean reads were assembled to contigs using metaSPAdes (v 3.13.2) (Bankevich et al., 2012) with k-mer sizes of 21, 33, 55, 77, 99, and 127. The open reading frames (ORFs) of the contigs were predicted using Prodigal (v 2.6.3) (Hyatt et al., 2010) and were searched against KEGG (Aramaki et al., 2019) to obtain the corresponding KO numbers. The denitrification genes and corresponding KO numbers were as follows: *nap*A, K02567; *nir*S, K15864; *nir*K, K00368; *nos*Z, K00376. The *nos*Z gene has only one KO number but includes *nos*Z I and *nos*Z II genes. We first obtained the protein sequence of *nos*Z gene and searched against the NCBI database.^1^ The *nos*Z I and *nos*Z II genes were distinguished based on their top-10 hits. The protein sequences of the ORFs were annotated using Kraken 2 (v 2.0.8b) (Wood et al., 2019) and GTDB combined (Parks et al., 2018; Chaumeil et al., 2019). The normalized abundance of ORF for all samples, i.e., the expression levels measured on the transcripts per million (TPM) scale, were estimated using Salmon (v 1.1.0) (Patro et al., 2017). Raw metagenomic sequencing data were deposited in the NCBI under BioProject PRJNA957066.

### 2.7. Statistical analysis

Redundancy analysis (RDA) was performed using Canoco 5 (v 5.0) and linear regression analysis, and graphs were generated using Graphism (v 8.0). The spearman correlation analysis between physiochemical properties and the abundance of denitrification genes was conducted by SPSS (v 26.0). *P*-value less than 0.05 (*p* < 0.05) was considered statistically significant.

## 3. Results

### 3.1. Physiochemical properties of paddy fields

The physiochemical properties of the paddy soils are shown in Table 1. The temperature at the time of sampling was relatively high, i.e., 31.3–36.7°C. The DO of paddy water was low and fluctuated in the range of 4.89 ± 0.02 to 13.02 ± 0.20 mg⋅L^–1^. The salinity value was two among all paddy water samples. The soil pH was weakly alkaline and its value ranged from 7.82 ± 0.03 to 9.34 ± 0.05. NH_4_^+^ was the main existing form of soil DIN, followed by NO_3_^–^. The NH_4_^+^ and NO_3_^–^ concentrations varied from 3.64 ± 0.19 to 9.72 ± 0.03 and 0.16 ± 0.02 to 5.94 ± 0.31 mg⋅kg^–1^, respectively. The NO_2_^–^ concentrations were low in soil samples, with a minimum value of 0.38 ± 0.01 mg⋅kg^–1^.

**TABLE 1:** Physiochemical characteristics of paddy soils.

Samples	Temperature (°C)	DO (mg⋅L^–1^)	Salinity	pH	NO_3_^–^ (mg⋅kg^–1^)	NO_2_^–^ (mg⋅kg^–1^)	NH_4_^+^ (mg⋅kg^–1^)
S1	33.8	4.89 ± 0.02	2	7.82 ± 0.03	5.94 ± 0.31	0.55 ± 0.03	7.88 ± 0.23
S2	33.1	8.09 ± 0.04	2	8.09 ± 0.05	3.86 ± 0.36	0.38 ± 0.01	9.72 ± 0.03
S3	32.5	8.06 ± 0.06	2	8.37 ± 0.03	0.16 ± 0.02	0.66 ± 0.03	6.03 ± 0.32
S4	31.3	7.96 ± 0.05	2	8.2 ± 0.16	0.38 ± 0.16	0.77 ± 0.04	6.76 ± 1.06
S5	34	11.84 ± 0.16	2	9.23 ± 0.07	1.51 ± 0.33	0.49 ± 0.16	3.64 ± 0.19
S6	31.9	8.66 ± 0.09	2	9.11 ± 0.06	1.37 ± 0.04	0.49 ± 0.01	5.01 ± 0.17
S7	36.7	13.02 ± 0.20	2	9.34 ± 0.05	0.88 ± 0.20	0.67 ± 0.09	3.92 ± 0.07

### 3.2. N_2_O concentration, ΔN_2_O concentration, N_2_O saturation, and N_2_O flux of air–water exchange

**FIGURE 2:**
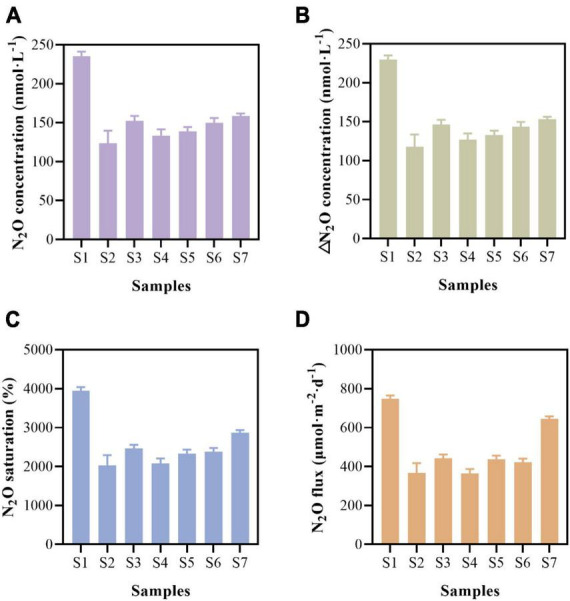
Dissolved nitrous oxide (N_2_O) concentration **(A)**, △N_2_O concentration **(B)**, N_2_O saturation **(C)**, and N_2_O emission flux **(D)** of paddy water.

Paddy water showed high N_2_O concentrations and saturations (Figure 2). The dissolved N_2_O concentration ranged from 123.65 ± 16.09 to 235.59 ± 5.59 nmol⋅L^–1^, with minimum and maximum values indicated by samples S2 and S1, respectively (Figure 2A). Excluding dissolved N_2_O at the air–water equilibrium, the ΔN_2_O concentration fluctuated between 117.56 ± 16.09 and 229.62 ± 5.59 nmol⋅L^–1^ (Figure 2B). The N_2_O dissolved in paddy water was supersaturated, with an average N_2_O saturation of 2588.13 ± 659.94% (Figure 2C). The paddy fields were a net source of atmospheric N_2_O, and the maximum N_2_O flux from the paddy water was estimated to be between 367.28 ± 50.26 and 747.03 ± 18.19 μmol⋅m^–2^⋅d^–1^ (Figure 2D).

### 3.3. Potential denitrification activities in surface paddy soils

**FIGURE 3:**
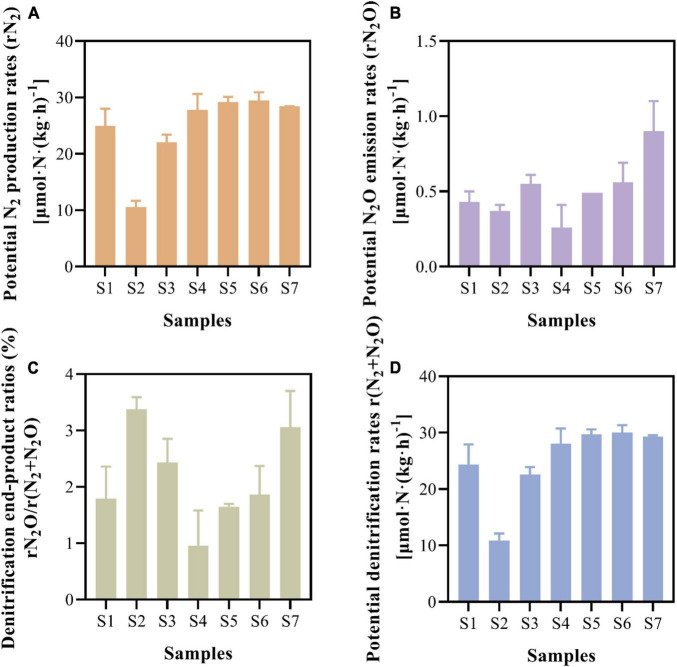
Potential N_2_ production rates **(A)**, potential N_2_O emission rates **(B)**, ratios of denitrification end-products **(C)**, and potential denitrification rates **(D)** of paddy soils.

The potential N_2_ production and N_2_O emission rates were measured via slurry incubation combined with ^15^N isotope tracer experiments (Figure 3). The paddy soils showed comparable N_2_ production rates, with an average rate of 24.63 ± 6.76 μmol⋅N⋅kg^–1^⋅h^–1^ (Figure 3A). The potential N_2_O emission rates fluctuated between 0.26 ± 0.15 and 0.90 ± 0.20 μmol⋅N⋅k⋅g^–1^⋅h^–1^, with an average value of 0.51 ± 0.20 μmol⋅N⋅kg^–1^⋅h^–1^ (Figure 3B). On average, the N_2_O emitted constituted 2.16 ± 0.85% of the denitrification end-products (Figure 3C), and the potential denitrification rates ranged from 10.92 ± 1.19 to 30.03 ± 1.33 μmol⋅N⋅kg^–1^⋅h^–1^ in paddy soils (Figure 3D).

### 3.4. Enzymatic activities of NOR and NOS and enzymatic activity ratios of NOR to NOS

**FIGURE 4:**
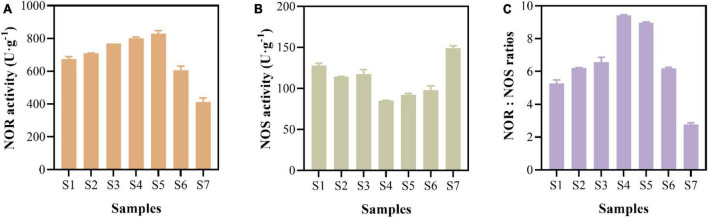
NO reductase (NOR) activity **(A)**, N_2_O reductase (NOS) activity **(B)**, and activity ratios of NOR to NOS **(C)** of paddy soils.

The enzymatic activities related to N_2_O production and N_2_O reduction were measured (Figure 4). The results showed that the activity of NOR was relatively high, ranging from 413.49 ± 23.84 to 829.52 ± 18.34 U⋅g^–1^ (Figure 4A). The activity of NOS fluctuated between 85.19 ± 0.32 and 148.92 ± 3.16 U⋅g^–1^ in the paddy soils (Figure 4B). The activity of NOR was 2.77 to 9.42 times than that of NOS, indicating that the production rate of N_2_O was higher than the N_2_O reduction rate at the enzymatic activity level (Figure 4C).

### 3.5. Abundance and abundance ratios of denitrification genes

**FIGURE 5:**
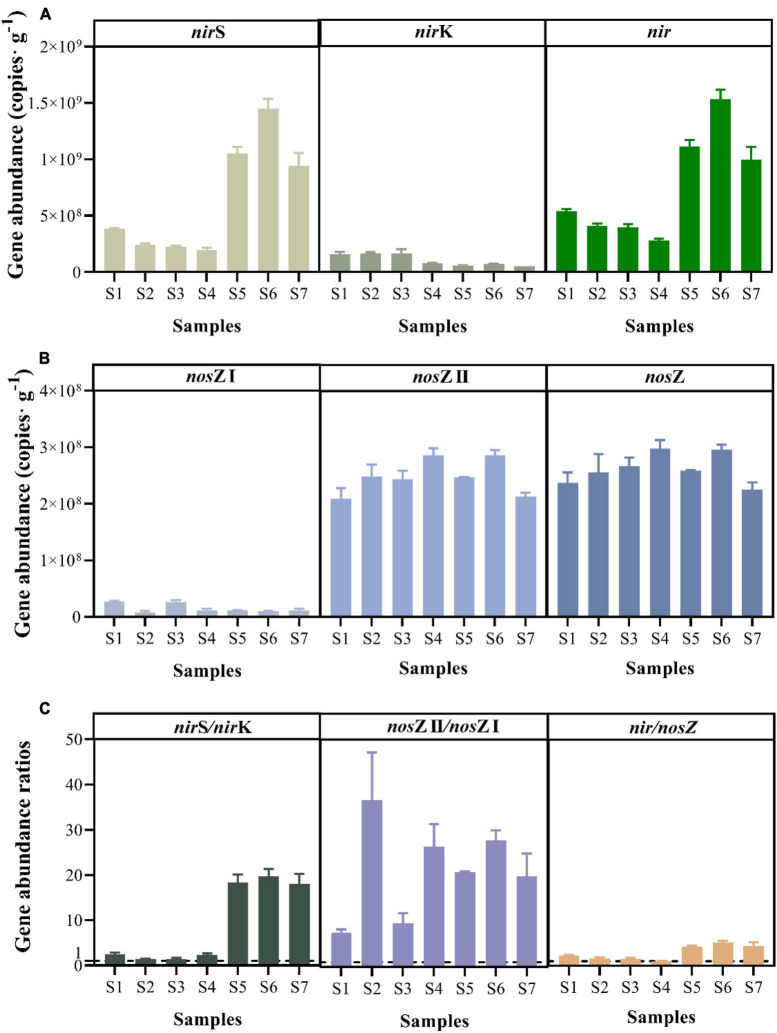
Abundance of key functional genes for denitrification, including *nir* (*nir*S and *nir*K) **(A)**, *nos*Z (*nos*Z I and *nos*Z II) **(B)**, and gene abundance ratios **(C)** of paddy soils.

The abundance of denitrification functional genes was successfully quantified in all paddy soils (Figure 5). The *nir* and *nos*Z genes include the *nir*S and *nir*K genes and *nos*Z I and *nos*Z II genes, respectively. The paddy soil showed higher abundance of the *nir* gene, with the *nir*S gene abundance ranging from (1.96 ± 0.20) × 10^8^ to (1.45 ± 0.09) × 10^9^ copies⋅g^–1^, and the *nir*K gene abundance ranging from (5.22 ± 0.02) × 10^7^ to (1.68 ± 0.35) × 10^8^ copies⋅g^–1^, respectively (Figure 5A). The abundance of the *nir*K gene was positive correlated with the NH_4_^+^ concentration (r = 0.786, *p* < 0.05) by the spearman correlation analysis. Moreover, the abundance of *nir* genes was positively correlated with the potential N_2_O emission rate (r = 0.786, *p* < 0.05). The abundance of the *nos*Z I and *nos*Z II genes fluctuated between (7.18 ± 0.29) × 10^6^ and (2.68 ± 0.13) × 10^7^ copies⋅g^–1^ and between (2.09 ± 0.19) × 10^8^ and (2.86 ± 0.13) × 10^8^ copies⋅g^–1^, respectively (Figure 5B). Based on the gene abundance ratios of *nir*S/*nir*K (average: 9.12 ± 9.00) and *nos*Z II/*nos*Z I (average: 21.27 ± 10.28), the *nir*S and *nos*Z II genes were the most abundant in paddy soils (Figure 5C). The abundances of *nos*Z I and *nos*Z II genes ranged from 10^8^ to 10^9^ and 10^8^, respectively (Figures 5A, B). The gene abundance ratios of *nir*/*nos*Z [which ranged from 0.94 to 5.17 (Figure 5C)] exceeded 1, except in sample S4.

### 3.6. Community composition of denitrification genes

**FIGURE 6:**
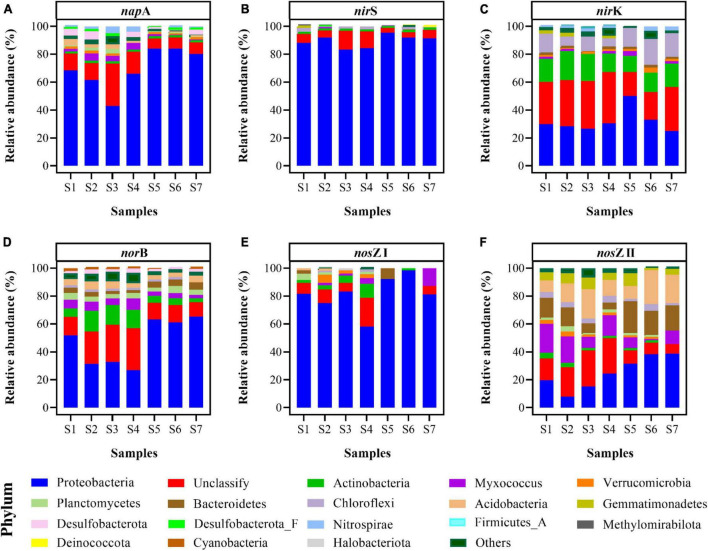
Community compositions of key functional genes for denitrification, including *nap*A **(A)**, *nir*S **(B)**, *nir*K **(C)**, *nor*B **(D)**, *nos*Z I **(E)**, and *nos*Z II **(F)** at the phylum level of paddy soils.

In total, 395,500,000 high-quality sequences were obtained after quality control (Supplementary Table 2). At the phylum level, the prokaryotic communities mainly belonged to Proteobacteria (27.87–41.8%), Actinobacteria (19.14–29.43%), unclassified bacteria (13.63–15.77%), Chloroflexi (2.73–5.06%), and Acidobacteria (2.37–4.32%) (Supplementary Figure 1). Community composition and diversity based on denitrification genes (including *nap*A, *nir*S, *nir*K, *nor*B, *nos*Z I, and *nos*Z II) were analyzed (Figure 6).

The *nap*A gene, catalyzing the reduction of NO_3_^–^ to NO_2_^–^, was abundant in Proteobacteria. The mean abundance of Proteobacteria of the *nap*A gene was 21.22 ± 15.38 TPM (Supplementary Table 3), which constituted 69.58 ± 14.78% of the *nap*A gene community (Figure 6A). Additionally, unclassified bacteria constituted 13.39 ± 8.06% of the *nap*A gene sequences, and the *nap*A gene distributed in Desulfobacterota and Myxococcus for some samples (Figure 6A).

The enzymes encoded by the *nir*S and *nir*K genes contributed to the reduction of NO_2_^–^ to NO. Similar to the composition of the *nap*A gene, a large proportion of the *nir*S gene appeared in Proteobacteria (83.33–95.39%), and a small proportion of unclassified bacteria (3.34–13.31%) was detected in all paddy soils. The abundances of Proteobacteria and unclassified bacteria in the *nir*S gene sequences ranged from 9.85 to 59.86 TPM and from 0.71 to 3.47 TPM, respectively (Supplementary Table 4). Additionally, Chloroflexi (0.14–1.89 TPM) and Actinobacteria (0.06–0.33 TPM) were observed in all samples (Figure 6B). The *nir*K gene was mainly distributed in unclassified bacteria (17.23–34.27%), Proteobacteria (25.05–49.96%), Actinobacteria (11.82–20.80%), and Chloroflexi (7.60–18.42%) (Figure 6C), and their abundances were 9.12 ± 5.40, 8.90 ± 3.40, 4.89 ± 2.73, and 3.27 ± 1.12 TPM, respectively (Supplementary Table 5). Besides, the community composition of the *nir*K gene was positively correlated with the potential N_2_O emission (r = 0.821, *p* < 0.05).

The *nor*B gene, catalyzing the reduction of NO to N_2_O, was abundant in Proteobacteria, unclassified bacteria, and Actinobacteria. Their mean abundances were 21.77 ± 46.32, 7.24 ± 24.72, 2.14 ± 12.61 TPM, respectively (Supplementary Table 6), and they constituted 26.74–65.35, 10.22–30.36, and 3.03–14.77% of the *nor*B gene sequences, respectively (Figure 6D). In addition, Myxococcus and Acidobacteria, which contained the *nor*B gene, were detected in all soil samples.

N_2_O reductase, encoded by the *nos*Z I and *nos*Z II genes, reduces N_2_O to N_2_. Proteobacteria (58.14–98.14%) was the main phylum of the *nos*Z I gene (Figure 6E), and its abundance fluctuated between 2.54 and 7.93 TPM (Supplementary Table 7). Only a few unclassified bacteria (0.00–20.57%) and Actinobacteria (0.00–10.05%) were indicated in the *nos*Z I gene sequence (Figure 6E). Meanwhile, for the *nos*Z II gene, the community composition was more diverse (Figure 6F). Proteobacteria was the dominant phylum; its abundance ranged from 2.04 to 13.21 TPM (Supplementary Table 8) and it constituted 7.97–38.67% of the relative abundance (Figure 6F). Other phyla, including Acidobacteria (8.01–24.36%), unclassified bacteria (7.00–25.95%), Bacteroidetes (4.92–22.84%), Myxococcus (2.07–20.99%), Gemmatimonadetes (1.05–9.28%), and Chloroflexi (0.75–4.78%), were observed in all soil samples (Figure 6F).

In general, Proteobacteria was the most typical group among the denitrification genes, and the relative abundance of classes in Proteobacteria based on the denitrification genes was analyzed (Supplementary Figure 2). Gammaproteobacteria was the dominant Proteobacteria in *nap*A (Supplementary Figure 2A), *nir*S (Supplementary Figure 2B), and *nor*B genes (Supplementary Figure 2D). Proteobacteria containing *nir*K (Supplementary Figure 2C), *nos*Z I (Supplementary Figure 2E), and *nos*Z II genes (Supplementary Figure 2F) were mainly composed of Alphaproteobacteria and Gammaproteobacteria.

The results of RDA showed that pH of the paddy soils significantly affected the community composition of most denitrification genes, including *nap*A, *nir*S, *nir*K, *nos*Z I, and *nos*Z II genes (*p* < 0.05) (Supplementary Figure 3). In addition, the community composition of the *nir*S gene was affected by the NO_3_^–^ concentration (*p* < 0.05) (Supplementary Figure 3A).

## 4. Discussion

### 4.1. Contribution of denitrification to N_2_O emission

Soil and water in paddy fields are closely related but rarely investigated simultaneously. In this study, we determined the N_2_O emission capacity of both paddy soil and paddy water. The potential N_2_O emission rates fluctuated between 0.26 ± 0.15 and 0.90 ± 0.20 μmol⋅N⋅kg^–1^⋅h^–1^ (Figure 3B), which was higher than those of soils from Hebei Province (0.029 μmol⋅N⋅kg^–1^⋅h^–1^) (Zhao et al., 2019) and similar to those of sediments from the Chongming Dongtan wetland (0.21–0.84 μmol⋅N⋅kg^–1^⋅h^–1^) (Gao et al., 2022). Based on the potential N_2_O emission rate in surface paddy soils, the potential N_2_O emission by denitrification reached 0.31 ± 0.12 mol N_2_O⋅m^–2^⋅yr^–1^, generating an emission estimation of 13.67 ± 5.44 g N_2_O⋅m^–2^⋅yr^–1^ in surface paddy soils (estimation method supplied in Supplementary material).

The concentration of N_2_O in the paddy water was high, i.e., 123.54 to 235.59 nmol⋅L^–1^ (Figure 2A). The concentration of N_2_O in the paddy water was approximately 18.17 times that in Indian estuaries (Rao and Sarma, 2013) and 7.72–14.72 times that in the Jinshui River (Zhao and Zhang, 2021). Excessive application of nitrogen fertilizer in paddy fields can promote the release of a significant amount of N_2_O (Gupta et al., 2021). As expected, supersaturation was observed in the paddy water, and its average N_2_O saturation (2588.13 ± 659.94%) was significantly higher than that of water from the Jinshui and Qi Rivers (Zhao and Zhang, 2021) and from the Shanghai River (770%) (Yu et al., 2013). The mean N_2_O flux (489.59 ± 147.28 μmol⋅m^–2^⋅d^–1^) in the paddy water were significantly higher than that in the upper Pearl River estuarine water (313 ± 150 μmol⋅m^–2^⋅d^–1^) (Lin et al., 2016) and the Shanghai city river (140 μmol⋅m^–2^⋅d^–1^) (Yu et al., 2013). Based on the N_2_O flux, the average N_2_O release reached 0.18 ± 0.05 mol⋅N_2_O m^–2^⋅yr^–1^, which generated an emission estimation of 7.86 ± 2.37 g N_2_O⋅m^–2^⋅yr^–1^ in the paddy water (estimation method supplied in Supplementary material). The N_2_O released from the surface paddy soils contributed to the N_2_O dissolved in the paddy water and was further converted, and the remaining N_2_O was released from the paddy water into the atmosphere.

### 4.2. Abundance of denitrification genes and enzymatic activities related to N_2_O production and reduction in paddy soil

Microbial denitrification is a four-step reduction process catalyzed by different enzymes, which are mainly encoded by the *nap*A, *nir*K/S, *nor*B, and *nos*ZI/II genes (Zumft, 1997). Both functional genes and enzymatic activities are vital to denitrification. N_2_O is an intermediate product of the denitrification process, and the relative contributions of N_2_O production and reduction determine whether N_2_O is released into the atmosphere as well as the amount released (Saarenheimo et al., 2015).

Regarding the abundance of the denitrification genes, previous studies pertaining to paddy soils (Zhao et al., 2019; Zhang et al., 2021) and wetland soils (Jiang et al., 2020) showed higher abundance of the *nir*S gene than the *nir*K gene. Similar to these studies, the abundance of *nir*S gene in the paddy soils was 1.39–19.71 times higher than that of *nir*K gene (Figures 5A, C). Furthermore, it was consistent with the better adaptation of the *nir*S gene to stable and watery environments compared with the *nir*K gene (Petersen et al., 2012). In addition, the abundance ratio of *nir*S to the 16S rRNA gene was positively correlated with the potential N_2_O emission rate (Supplementary Figure 4), as similarly observed in soils (Assémien et al., 2019; Zhao et al., 2019). Among the genes contributing to N_2_O reduction, the abundance of *nos*Z II gene was higher than that of *nos*Z I gene, and the abundance ratios of *nos*Z II to *nos*Z I genes ranged from 2.67 to 10.91 (Figures 5B, C). The ratios were consistent with the typically reported value ranging between 1.5 and 10 in different environments (Jones et al., 2013; Frame et al., 2014; Tsiknia et al., 2015). Additionally, previous studies demonstrated that the abundance of the *nos*Z II gene contributed significantly to the soil N_2_O sink capacity (Jones et al., 2014). Thus, *nos*Z II gene may be vital to N_2_O reduction. Considering genes related to N_2_O production and reduction, the *nir*/*nos*Z abundance ratios ranged from 0.94 to 5.17 (Figure 5C), which were similar to those in farmland soil (Zhao et al., 2019) and lake sediments (Saarenheimo et al., 2015). The ratios revealed an imbalance between N_2_O production and reduction, indicating that the potential of paddy soils to produce N_2_O is greater than reduce it at the genetic level (Domeignoz-Horta et al., 2015).

Microbial denitrification is an enzyme-mediated biochemical process; however, the activities of denitrifying enzymes have been disregarded in most researches (Morales et al., 2010; Petersen et al., 2012). Some studies showed that enzymatic activity affected sediment N_2_O emissions (Zheng et al., 2014; Su et al., 2019). NOR and NOS are enzymes that catalyze the production and reduction processes of N_2_O, respectively (Braker and Tiedje, 2003; Zumft and Kroneck, 2007). In our study, the NOR activity in paddy soil (413.19–829.52 U⋅g^–1^) was higher than that in Donghu sediment (274.70 U⋅g^–1^), while the NOS activity (85.19–148.92 U⋅g^–1^) was lower than that in Donghu sediment (188.73 U⋅g^–1^) (Zhang et al., 2022). NOR activity was higher than NOS activity, with the ratio of NOR to NOS ranging from 2.77 to 9.42 (Figure 4). This ratio was higher than the ratio detected in Donghu Lake sediments, i.e., 1.46 (Zhang et al., 2022), and that determined from riparian sediments, i.e., 0.33 (Su et al., 2019). These enzymatic activities agreed well with the genetic potential of N_2_O emissions (Figure 5C) and potential N_2_O emission rates (Figure 3C) in the paddy soils. The enzymatic activity ratios suggest that paddy soils have a higher potential to produce N_2_O than to reduce it at the protein level. Enzyme are proteins encoded by functional genes, in view of the fact that the gene abundance of *nir* was higher than that of *nos*Z gene (Figure 5C), the higher activity of NOR than NOS was more likely to be the result of gene expression.

### 4.3. Modularity of denitrification process and taxonomy groups of different denitrifying bacteria

By performing metagenomic sequencing, we achieved a more comprehensive understanding of the bacterial community composition in paddy soils, particularly that of denitrifying bacteria. Bacteria in the paddy soils investigated were mainly Proteobacteria, Actinobacteria, Chloroflexi, and Acidobacteria at the phylum level (Supplementary Figure 1); such microbial community composition has been similarly detected in paddy soils from Jiangxi Province (Zhang et al., 2021). However, the dominant community composition varied for different denitrification genes (Figure 6).

**FIGURE 7:**
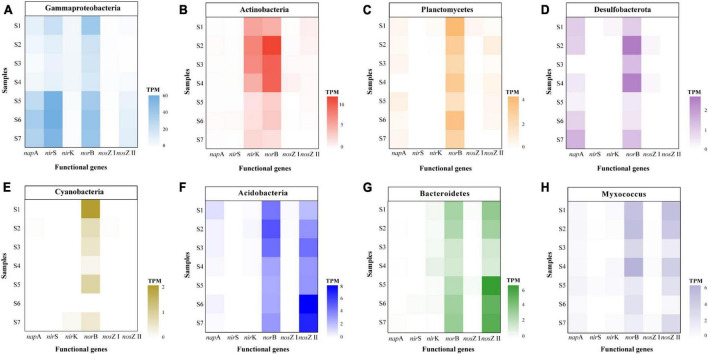
Abundance of key functional genes for denitrification in dominant phyla of *nor*B gene, including Gammaproteobacteria **(A)**, Actinobacteria **(B)**, Planctomycetes **(C)**, Desulfobacterota **(D)**, Cyanobacteria **(E)**, Acidobacteria **(F)**, Bacteroidetes **(G)**, and Myxococcus **(H)**.

Several genes including *nap*A, *nir*S, *nir*K, and *nor*B are closely related with the production of N_2_O (Zumft, 1997). Our results showed that microbial groups with different denitrification genes exhibited distinct taxonomic characteristics. Proteobacteria was the most abundant phylum for the *nap*A gene in paddy soils, which was consistent with the results of sediments from the Pearl River Estuary (Wang et al., 2021). In addition, other *nap*A-harboring bacteria were affiliated with Myxococcus and Actinobacteria (Figure 6A and Supplementary Table 3). Previous studies based on high-throughput amplicon sequencing or clone libraries demonstrated that the *nir*S gene belonged to Proteobacteria in sediments (Liu et al., 2020), red soil (Ye et al., 2022) and paddy soil (Yoshida et al., 2009). We performed metagenomic analysis, which avoided primer preference, and discovered that the *nir*S gene predominantly existed in Proteobacteria, but also in a small proportion of unclassified bacteria, Chloroflexi, and Actinobacteria in all the paddy soils (Figure 6A and Supplementary Table 4). The community diversity of *nir*S gene in this study was higher than that reported in other studies (Jiao et al., 2018; Liu et al., 2020; Ye et al., 2022). However, the community composition of the *nir*K gene was more diverse than that of *nir*S gene. The sequences of *nir*K gene were assigned to unclassified bacteria, Proteobacteria, Actinobacteria, and Chloroflexi (Figure 6C and Supplementary Table 5). Similarly, Proteobacteria and Chloroflexi were the dominant phyla of the *nir*K gene in sediments from the Pearl River Estuary (Wang et al., 2021). Owing to primer limitations, information regarding groups containing the *nor*B gene is limited. In this study, *nor*B gene was widespread in Proteobacteria, unclassified bacteria, Actinobacteria, Myxococcus, Acidobacteria, Planctomycetes, and Bacteroidetes (Figure 6 and Supplementary Table 6). Moreover, Gammaproteobacteria contained high levels of both the *nir*S gene (9.22–57.61 TPM) and *nor*B gene (16.58–42.58 TPM) (Figure 7A). This result was similar to a previous finding where a high percentage of organisms with the *nir*S gene was discovered among bacteria that also contained the *nor*B gene (Graf et al., 2014). However, the other groups of microorganisms did not contain the *nir*S gene but contained the *nor*B gene (Figure 7). Many dominant phyla of the *nor*B gene (including Actinobacteria, Planctomycetes, Desulfobacterota, Cyanobacteria, Acidobacteria, Bacteroidetes, and Myxococcus) do not have or have a relatively low abundance of the *nir*S gene because of the predominant distribution of the *nir*S gene in the Proteobacteria phylum (Figure 7).

The reduction of N_2_O, which is the only pathway for the N_2_O sink, is solely related to *nos*Z I and *nos*Z II genes (Jones et al., 2013). Although the *nos*Z I gene was predominately related to Proteobacteria (Green et al., 2010; Xiang et al., 2022), many previously overlooked phyla were identified in all paddy soils, including Actinobacteria and Myxococcus (Figure 6C and Supplementary Table 7). Thus, previous community studies based on high-throughput amplicon sequencing of *nir*S and *nos*Z I genes inevitably overlooked some denitrifying bacterial diversity. The *nos*Z II gene appeared in Proteobacteria, Acidobacteria, unclassified bacteria, Bacteroidetes, Myxococcus, Gemmatimonadetes, and Chloroflexi in all the paddy soils investigated (Figure 6D and Supplementary Table 8); similarly, these phyla of the *nos*Z II gene have been reported in sediments from Bohai Sea and Jiulong River (Dai et al., 2022). For the *nos*Z I and *nos*Z II genes, Gemmatimonadetes appeared only in the *nos*Z II gene (Supplementary Tables 7, 8), as detected in soils from Hebei Province (Zhao et al., 2019). In addition, the abundance of *nos*Z II was higher than that of *nos*Z I in many phyla, including Acidobacteria, Bacteroidetes, Myxococcus, and Chloroflexi (Supplementary Tables 7, 8), indicating that these phyla are more likely to be NosZ-II-type N_2_O-reducing bacteria.

*Nap*A, *nir*S, and *nos*Z I genes were mainly affiliated with Proteobacteria, whereas *nir*K, *nor*B, *nos*Z II genes were composed of different phyla (Figure 6). As the *nor*B gene contributes to N_2_O production (Zumft, 1997), we further analyzed the abundance of denitrification genes in the dominant community of the *nor*B gene. Gammaproteobacteria and many phyla, including Actinobacteria, Planctomycetes, Desulfobacterota, Cyanobacteria, Acidobacteria, Bacteroidetes, and Myxococcus, harbored the *nor*B gene and did not have or had a relatively lower abundance of the *nos*Z gene (Figures 7A–E). This imbalance between N_2_O production and the reduction in these phyla may have contributed to the release of N_2_O from paddy soils. In addition, the abundance of the *nor*B and *nos*Z II genes was relatively high in Acidobacteria, Bacteroidetes, and Myxococcus, whereas the abundance of *nap*A, *nir*K, and *nos*Z I genes was low, and that of *nir*S gene was absent (Figures 7F–H). This suggests that Acidobacteria, Bacteroidetes, and Myxococcus ware more likely to be NosZ-II-type N_2_O-reducing bacteria that contain the *nor*B gene but lack the *nir* gene. This was consistent with the discovery of bacteria that contain the *nos*Z gene and lack the *nir* gene, mainly in Bacteroidetes (Graf et al., 2014), and further extended the understanding that Bacteroidetes did not contain the *nir* gene but harbored the *nor*B and *nos*Z II genes. For Alphaproteobacteria, the similar abundance distribution of the *nor*B gene and the *nos*Z I gene indicate that they possess both N_2_O production and reduction potential (Supplementary Figure 5). It is inferred that their contribution to N_2_O emissions is likely smaller than that of Gammaproteobacteria. Results of co-occurrence analysis between different denitrification genes showed that denitrification in paddy soil was a highly modular and truncated process that caused an imbalance in the N_2_O production and reduction processes, thus resulting in N_2_O emission. However, more data regarding the distribution of denitrification genes in diverse microbial taxa are required to obtain a more comprehensive understanding of the modularity in denitrification.

## 5. Conclusion

This study provides valuable insights into N_2_O emission during paddy soil denitrification. The high potential for N_2_O emission was facilitated by the imbalance between N_2_O production and reduction processes in terms of gene abundance (*nir*/*nos*Z) and enzymatic activity (NOR/NOS). The *nir*S and *nos*Z II genes were abundant in paddy soils. Furthermore, the composition of denitrification genes demonstrated a highly modularized denitrification process in paddy fields. Gammaproteobacteria and other phyla, including Actinobacteria, Planctomycetes, Desulfobacterota, Cyanobacteria, Acidobacteria, Bacteroidetes, and Myxococcus, containing the *nor*B gene without *nos*Z genes, may contribute to N_2_O emission from paddy soils. These results enhance our understanding of N_2_O emission during denitrification and provide a theoretical basis for mitigating greenhouse gas emissions in agricultural ecosystems.

## Data availability statement

The datasets presented in this study can be found in online repositories. The names of the repository/repositories and accession number(s) can be found in below: https://www.ncbi.nlm.nih.gov/, PRJNA957066.

## Author contributions

HX conducted the experiments and wrote the original draft. HX, YH, and AL reviewed and edited the manuscript. HX, JW, YW, FY, JY, and JL performed field sampling. YH and AL funded this study. All authors have read and approved the final version of the manuscript.

## Footnotes

1
https://ftp.ncbi.nlm.nih.gov/blast/db/
