# Supplementary material for: Denitrification contributes to N2O emission in paddy soils
Source: Front Microbiol. 2023 Jun 16;14:1218207. doi: 10.3389/fmicb.2023.1218207 (PMC10313071; doi:10.3389/fmicb.2023.1218207)
Supplement: Supplementary file 1 [file Data_Sheet_1.pdf]

Supporting Information for

Denitrification contributes to N_2O emission in paddy soils

Hua Xiang[1,2,3], Yiguo Hong[2]*, Jiapeng Wu[2], Yu Wang[2], Fei Ye[2], Jiaqi Ye[2], Jing Lu[2], Aimin Long[1,3]*

[1]State Key Laboratory of Tropical Oceanography (LTO), South China Sea Institute of Oceanology, Chinese Academy of Sciences. Guangzhou 510301, P.R China

[2]Institute of Environmental Research at Greater Bay Area, Key Laboratory for Water Quality and Conservation of the Pearl River Delta, Ministry of Education, Guangzhou University, Guangzhou 510006, P.R China

[3]University of Chinese Academy of Sciences, Beijing 100049, P.R China

Corresponding authors:

*Yiguo Hong: yghong@gzhu.edu.cn

*Aimin Long: longam@scsio.ac.cn

Contents of this file

Figure S1 to S5

Table S1 to S8

Figure Captions

Figure S1 Community composition of paddy soils revealed by metagenome. Metagenomic reads were annotated by Karken2, and the phyla with top 10 abundance were shown.

Figure S2 Relative abundance of different classes in Proteobacteria based on denitrification genes, including the *nap*A (a), the *nir*S (b), *nir*K (c), the *nor*B (d), the *nos*Z I (e), and the *nos*Z II (f).

Figure S3 Redundancy analysis (RDA) based on physiochemical characteristics and denitrifying gene community composition in paddy soils. The community composition of denitrification functional genes includes the *nap*A (a), the *nir*S (b), the *nir*K (c), the *nor*B (d), the *nos*Z I (e), and the *nos*Z II (f). The environmental factors indicated by red lines show that they can significantly affect the community composition (p<0.05).

Figure S4 Linear regression between the gene abundance ratios of *nir*S to 16S and potential N_2O emission rates in paddy soils.

Figure S5 Abundance of key functional genes for denitrification in Alphaproteobacteria.

Table Captions

Table S1 Primers and PCR amplification protocols in this study.

Table S2 Sequencing statistics of the paddy soil samples after quality control.

Table S3 Community composition and abundance of top 10 phyla of the *nap*A gene.

Table S4 Community composition and abundance of the 9 phyla of the *nir*S gene.

Table S5 Community composition and abundance of top 10 phyla of the *nir*K gene.

Table S6 Community composition and abundance of top 10 phyla of the *nor*B gene.

Table S7 Community composition and abundance of top 10 phyla of the *nos*Z I gene.

Table S8 Community composition and abundance of top 10 phyla of the *nos*Z II gene.

Estimation methods

Estimation method I: The calculating method of the N_2O release from paddy water.

Estimation method II: The calculating method of the N_2O release potential by denitrification in paddy soils.

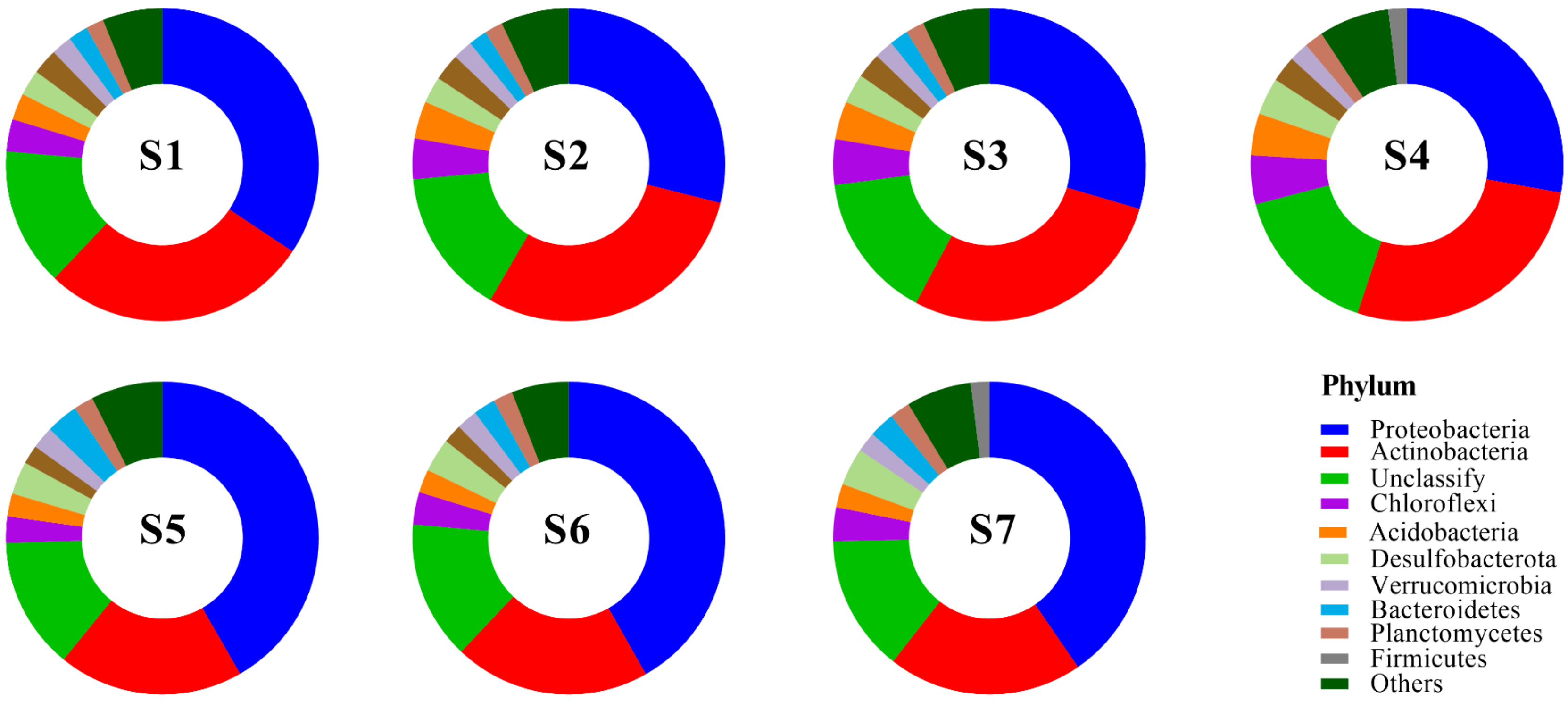

Figure S1 Community composition of paddy soils revealed by metagenome. Metagenomic reads were annotated by Karken2, and the phyla with top 10 abundance were shown.

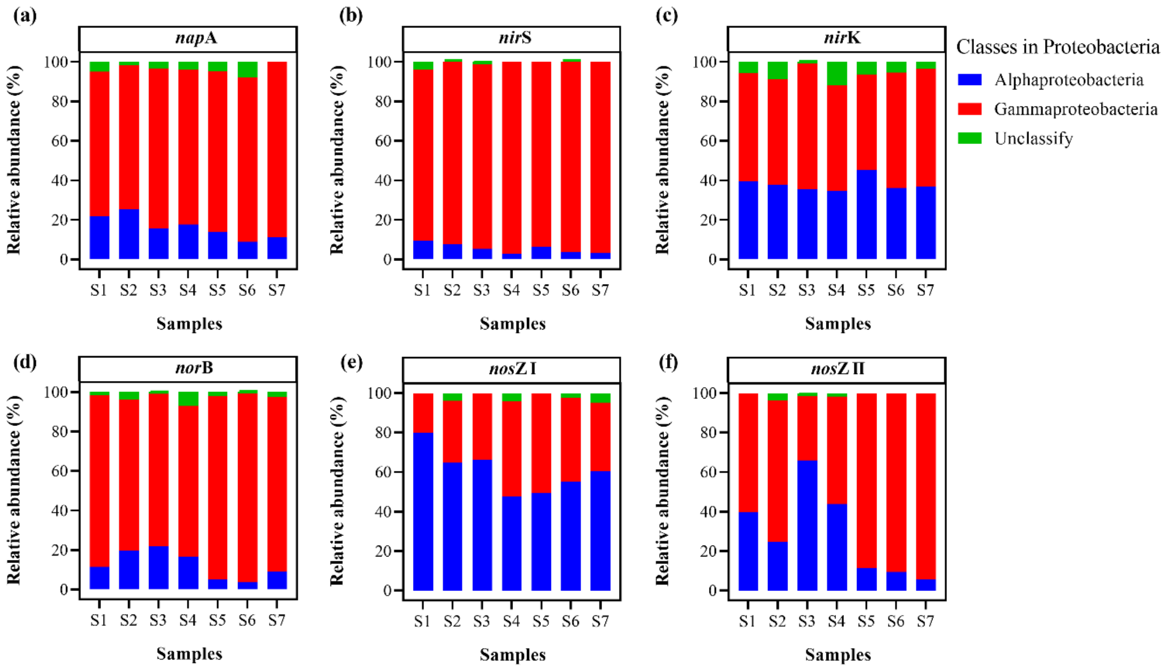

Figure S2 Relative abundance of different classes in Proteobacteria based on denitrification genes, including the *nap*A (a), the *nir*S (b), *nir*K (c), the *nor*B (d), the *nos*Z I (e), and the *nos*Z II (f).

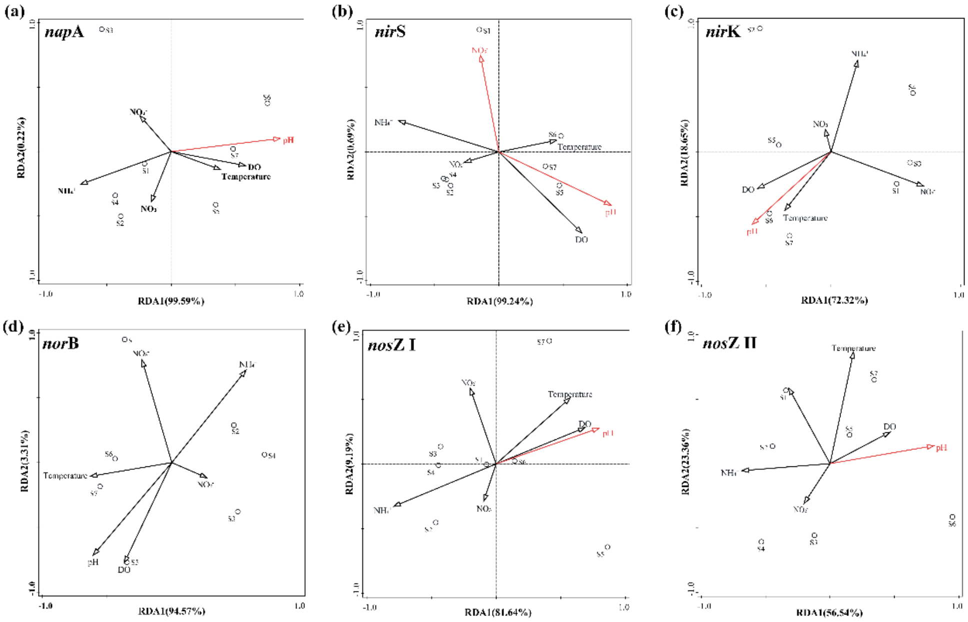

Figure S3 Redundancy analysis (RDA) based on physiochemical characteristics and denitrifying gene community composition in paddy soils. The community composition of denitrification functional genes includes the *nap*A (a), the *nir*S (b), the *nir*K (c), the *nor*B (d), and the *nos*Z I (e), and the *nos*Z II (f). The environmental factors indicated by red lines show that they can significantly affect the community composition (p<0.05).

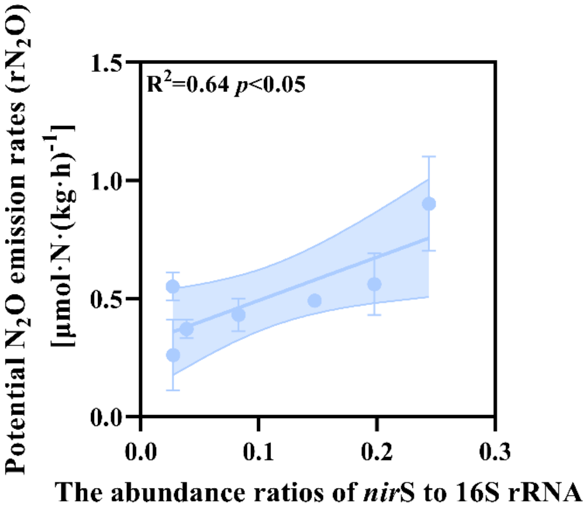

Figure S4 Linear regression between the gene abundance ratios of *nir*S to 16S and potential N_2O emission rates in paddy soils.

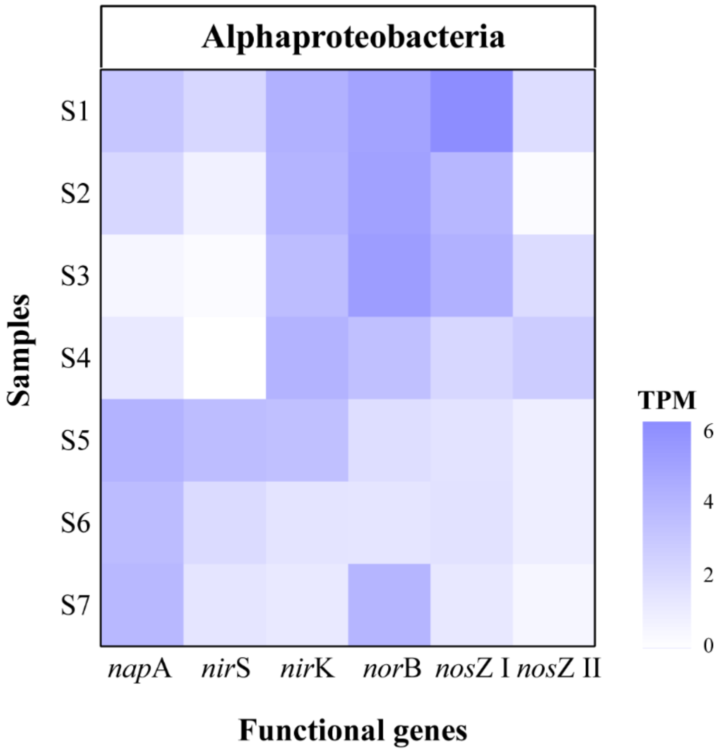

Figure S5 Abundance of key functional genes for denitrification in Alphaproteobacteria.

Table S1 Primers and qPCR amplification protocols in this study.

Gene	Primer pair	Sequences (5'-3')	Amplicon (bp)	PCR condition	Reference
**Bacterial 16S**	515F<br>806R	GTGYCAGCMGCCGCGGTAA<br>GGACTACNVGGGTWTCTAA	291	5 min at 94°C, 40 cycles consisting of 30 s at 94°C, 45s at 57°C, 45s at 72°C.	(Caporaso et al., 2011)
***nir*S**	cd3aF<br>R3cd	GTSAACGTSAAGGARACSGG<br>GASTTCGGRTGSGTCTTGA	425	3 min at 95°C, 40 cycles consisting of 45 s at 95°C, 45 s at 58°C and 40 s at 72°C.	(Throback et al., 2004; Yergeau et al., 2007)
***nir*K**	F1aCu<br>R3Cu	ATCATGGTSCTGCCGCG<br>TTGGTGTTRGACTAGCTCCG	474	2 min at 95°C, 36 cycles consisting of 30 s at 95°C, 45 s at 55°C and 45 s at 72°C.	(Hallin and Lindgern, 1999)
***nosZ* I**	*nos*Z 2F<br>*nos*Z 2R	CGCRACGGCAASAAGGTSMSSGT<br>CAKRTGCAKSGCRTGGCAGAA	267	5 min at 95°C, 35 cycles consisting of 30 s at 95°C, 1 min at 68°C and 1 min at 72°C, 10 min at 72°C.	(Henry et al., 2006)
***nosZ* II**	*nos*Z IIF<br>*nos*Z IIR	CTIGGICCIYTKCAYAC<br>GCIGARCARAAITCBGTRC	690-720	2 min at 95°C, 40 cycles consisting of 15 s at 95°C, 1 min at 60°C and 1 min at 72°C.	(Jones et al., 2013)

Table S2 Sequencing statistics of the paddy soil samples after quality control.

Sample	Sequence	Base	Q20(%)[a]	Q30(%)[b]
**S1**	3.48E+07	1.03E+10	98.809	96.0703
**S2**	1.84E+08	5.45E+10	98.7057	95.7343
**S3**	3.71E+07	1.10E+10	98.7556	95.8637
**S4**	3.80E+07	1.13E+10	98.7132	95.7308
**S5**	3.18E+07	9.40E+09	98.7889	95.9765
**S6**	3.08E+07	9.11E+09	98.7448	95.8445
**S7**	3.90E+07	1.15E+10	98.7175	95.7395

[a] *The percentage of bases with a sequencing error rate of less than 1%*

[b] *The percentage of bases with a sequencing error rate of less than 0.1%*

Table S3 Community composition and abundance of top 10 phyla of the *nap*A gene.

***nap*A**	**Proteobacteria**	**Unclassify**	**Desulfobacterota**	**Myxococcus**	**Planctomycetes**	**Nitrospirae**	**Acidobacteria**	**Desulfobacterota_F**	**Verrucomicrobia**	**Actinobacteria**	**Others**
**S1**	15.02	2.65	1.00	0.68	0.46	0.17	1.00	0.26	0.11	0.09	0.55
**S2**	9.51	1.86	0.91	0.80	0.33	0.33	0.40	0.22	0.31	0.26	0.50
**S3**	5.04	3.56	0.00	0.49	0.45	0.57	0.38	0.27	0.13	0.16	0.67
**S4**	8.23	1.97	0.46	0.69	0.00	0.49	0.21	0.00	0.00	0.08	0.34
**S5**	31.59	2.74	0.27	1.06	0.80	0.00	0.00	0.50	0.10	0.07	0.50
**S6**	43.56	4.15	0.88	0.42	0.33	0.44	0.39	0.86	0.38	0.26	0.19
**S7**	35.58	3.64	1.52	0.89	0.44	0.72	0.14	0.35	0.12	0.15	0.84

Table S4 Community composition and abundance of the 9 phyla of the *nir*S gene.

***nir*S**	**Proteobacteria**	**Unclassify**	**Chloroflexi**	**Actinobacteria**	**Deinococcota**	**Myxococcus**	**Gemmatimonadetes**	**Bacteroidetes**	**Methylomirabilota**	**Desulfobacterota_F**
**S1**	25.26	1.81	1.20	0.06	0.00	0.00	0.26	0.00	0.06	0.00
**S2**	12.92	0.71	0.14	0.11	0.05	0.07	0.00	0.00	0.02	0.03
**S3**	9.85	1.57	0.32	0.07	0.00	0.00	0.00	0.00	0.00	0.00
**S4**	11.08	1.57	0.30	0.19	0.00	0.00	0.00	0.00	0.00	0.00
**S5**	59.45	2.08	0.36	0.09	0.00	0.34	0.00	0.00	0.00	0.00
**S6**	59.86	2.52	1.89	0.33	0.15	0.00	0.00	0.19	0.00	0.00
**S7**	53.33	3.47	1.12	0.06	0.30	0.00	0.00	0.00	0.00	0.00

Table S5 Community composition and abundance of top 10 phyla of the *nir*K gene.

***nir*K**	**Unclassify**	**Proteobacteria**	**Actinobacteria**	**Chloroflexi**	**Verrucomicrobiota**	**Gemmatimonadetes**	**Nitrospirae**	**Bacteroidetes**	**Myxococcus**	**Firmicutes_A**	**Others**
**S1**	11.30	11.19	6.13	5.21	0.67	0.90	0.25	0.40	0.45	0.00	0.83
**S2**	13.30	11.33	8.34	3.05	0.59	1.05	0.29	0.30	0.25	0.15	1.44
**S3**	13.76	10.64	7.84	4.45	0.08	0.00	0.88	0.43	0.00	0.63	1.45
**S4**	14.93	12.43	5.40	2.40	0.59	0.67	0.27	0.91	0.59	0.16	2.35
**S5**	2.77	8.04	1.90	2.24	0.24	0.00	0.00	0.20	0.51	0.00	0.20
**S6**	2.85	4.72	2.00	2.64	0.51	0.00	0.45	0.29	0.00	0.00	0.86
**S7**	4.93	3.93	2.62	2.88	0.23	0.00	0.47	0.03	0.28	0.00	0.31

Table S6 Community composition and abundance of top 10 phyla of the *nor*B gene.

*nor*B	Proteobacteria	Unclassify	Actinobacteria	Myxococcus	Acidobacteria	Planctomycetes	Bacteroidetes	Desulfobacterota	Cyanobacteria	Chloroflexi	Others
**S1**	44.94	11.57	5.32	5.37	4.31	4.18	3.29	1.00	2.19	0.99	3.69
**S2**	26.72	19.89	12.61	5.70	5.44	3.03	2.94	2.63	0.74	0.93	4.77
**S3**	24.92	20.25	10.68	3.57	4.58	2.37	1.71	1.31	0.55	0.71	5.29
**S4**	21.77	24.72	10.56	6.70	2.87	3.22	1.60	2.50	0.20	1.15	6.12
**S5**	40.39	7.53	3.22	1.94	2.57	1.82	2.69	0.44	0.98	0.32	1.87
**S6**	44.46	9.00	3.58	2.62	2.71	3.70	3.49	0.52	0.00	0.91	1.72
**S7**	46.32	7.24	2.14	1.51	3.26	2.70	3.82	1.25	0.48	0.00	2.15

Table S7 Community composition and abundance of top 10 phyla of the *nos*Z I gene.

*nos*Z I	Proteobacteria	Unclassify	Actinobacteria	Myxococcus	Verrucomicrobia	Planctomycetes	Bacteroidetes	Chloroflexi	Acidobacteria	Halobacteriota	Others
**S1**	7.93	0.77	0.19	0.00	0.00	0.44	0.22	0.00	0.17	0.00	0.00
**S2**	6.37	0.84	0.24	0.16	0.49	0.05	0.00	0.19	0.07	0.05	0.04
**S3**	6.64	0.51	0.40	0.10	0.20	0.00	0.00	0.00	0.00	0.14	0.00
**S4**	5.01	1.77	0.87	0.35	0.25	0.12	0.00	0.17	0.00	0.00	0.08
**S5**	3.55	0.00	0.00	0.00	0.00	0.00	0.30	0.00	0.00	0.00	0.00
**S6**	3.34	0.00	0.06	0.00	0.00	0.00	0.00	0.00	0.00	0.00	0.00
**S7**	2.54	0.19	0.00	0.40	0.00	0.00	0.00	0.00	0.00	0.00	0.00

Table S8 Community composition and abundance of top 10 phyla of the *nos*Z II gene.

*nos*Z II	Proteobacteria	Acidobacteria	Unclassify	Bacteroidetes	Myxococcus	Gemmatimonadetes	Chloroflexi	Verrucomicrobia	Actinobacteria	Planctomycetes	Others
**S1**	5.20	2.12	4.18	4.02	5.56	1.61	1.17	0.70	1.02	0.16	0.75
**S2**	2.04	3.39	5.37	3.44	4.82	1.90	1.00	0.95	0.80	0.95	0.93
**S3**	3.26	4.53	5.54	1.62	1.72	1.75	0.73	0.21	0.32	0.23	1.45
**S4**	6.87	3.26	7.19	1.39	4.24	1.41	1.33	0.59	0.34	0.60	0.96
**S5**	10.31	3.32	3.14	7.48	2.66	3.04	0.24	0.51	0.37	0.50	1.17
**S6**	12.67	8.08	2.77	5.86	0.69	0.35	1.59	0.47	0.17	0.40	0.13
**S7**	13.21	6.96	2.39	6.19	3.28	1.48	0.52	0.00	0.00	0.00	0.14

Estimation method

Estimation method I: The calculating method of N_2O release according to N_2O flux from paddy water

The amount of N_2O release in paddy water was estimated according to the following equation:

$$N_{N2O\text{-}W}=F\times S\times T \quad (1)$$

where $N_{N2O\text{-}W}$ (mol m^{-2} yr^{-1}) denotes the annual N_2O release in paddy water, F (μmol m^{-2} d^{-1}) denotes the N_2O flux at the water-air interface, which was calculated in section 2.2 of the article, S denotes 1 m^2 of paddy water, T denotes the days in one year (365 days).

Estimation method II: calculating method of the N_2O release potential by denitrification in paddy soils

The amount of potentially N_2O released by denitrification in surface paddy soils was estimated according to the following equation:

$$N_{N2O\text{-}S}=rN_2O\times 0.5\times \rho\times s\times h\times t \quad (2)$$

where $N_{N2O\text{-}S}$ (mol $N_2O\cdot m^{-2}\cdot yr^{-1}$) denotes the annually amount of N_2O released by denitrification in surface paddy soils; rN_2O denotes potential N_2O emission rates (μmol N kg^{-1} h^{-1}); 0.5 is the conversion factor that converts N_2O-N to N_2O; ρ denotes the density of soils (1.40 $g\cdot cm^{-3}$) (Cai et al., 2022); s denotes 1 m^2 paddy soil (i.e., 1000 cm^2); h denotes the depth of surface paddy soils (10 cm); and t represents the hours in a year (8760 h).

Reference

Cai, M., Li, S., Ye, F., Hong, Y.G., Lu, M.Q., Op den Camp, H.J.M., Wang, Y. 2022. Artificial ponds as hotspots of nitrogen removal in agricultural watershed. Biogeochemistry 159(3), 283-301.

Caporaso, J., Lauber, C., Walters, W., Berg-Lyons, D., Lozupone, C., Turnbaugh, P., Fierer, N., Knight, R. 2011. Global patterns of 16S rRNA diversity at a depth of millions of sequences per sample. Proc. Natl. Acad. Sci. U. S. A. 108 Suppl 1, 4516-4522.

Hallin, S., Lindgern, P.-E. 1999. PCR detection of genes encoding nitrite reductase in denitrifying bacteria. Appl. Environ. Microbiol. 65(4), 1652-1657.

Henry, S., Bru, D., Stres, B., Hallet, S., Philippot, L. 2006. Quantitative detection of the *nos*Z gene, encoding nitrous oxide reductase, and comparison of the abundances of 16S rRNA, *nar*G, *nir*K, and *nos*Z genes in soils. Appl. Environ. Microbiol. 72(8), 5181-5189.

Jones, C.M., Graf, D.R., Bru, D., Philippot, L., Hallin, S. 2013. The unaccounted yet abundant nitrous oxide-reducing microbial community: a potential nitrous oxide sink. ISME J. 7(2), 417-426.

Throback, I.N., Enwall, K., Jarvis, A., Hallin, S. 2004. Reassessing PCR primers targeting *nir*S, *nir*K and *nos*Z genes for community surveys of denitrifying bacteria with DGGE. FEMS Microbiol. Ecol. 49(3), 401-417.

Yergeau, E., Kang, S., He, Z., Zhou, J., Kowalchuk, G. 2007. Functional microarray analysis of nitrogen and carbon cycling genes across an Antarctic latitudinal transect. ISME J. 1(2), 163-179.
